# Reduced-port totally robotic distal subtotal gastrectomy for gastric cancer: 100 consecutive cases in comparison with conventional robotic and laparoscopic distal subtotal gastrectomy

**DOI:** 10.1038/s41598-020-73118-9

**Published:** 2020-09-29

**Authors:** Won Jun Seo, Taeil Son, Hyejung Shin, Seohee Choi, Chul Kyu Roh, Minah Cho, Hyoung-Il Kim, Woo Jin Hyung

**Affiliations:** 1 Department of Surgery, Korea University College of Medicine, Seoul, Korea; 2 Department of Surgery, Yonsei University College of Medicine, 50 Yonsei-ro, Seodaemun-gu, Seoul, 120-752 Korea; 3 Gastric Cancer Center, Yonsei Cancer Center, Yonsei University Health System, Seoul, Korea; 4 Robot and MIS Center, Severance Hospital, Yonsei University Health System, Seoul, Korea; 5 Biostatistics Collaboration Unit, Department of Biomedical Systems Informatics, Yonsei University College of Medicine, Seoul, Korea; 6 Department of Surgery, Ajou University School of Medicine, Suwon, Korea

**Keywords:** Gastrointestinal diseases, Stomach

## Abstract

By overcoming technical difficulties with limited access faced when performing reduced-port surgery for gastric cancer, reduced-port totally robotic gastrectomy (RPRDG) could be a safe alternative to conventional minimally invasive gastrectomy. An initial 100 consecutive cases of RPRDG for gastric cancer were performed from February 2016 to September 2018. Short-term outcomes for RPRDG with those for 261 conventional laparoscopic (CLDG) and for 241 robotic procedures (CRDG) over the same period were compared. Learning curve analysis for RPRDG was conducted to determine whether this procedure could be readily performed despite fewer access. During the first 100 cases of RPRDG, no surgeries were converted to open or laparoscopic surgery, and no additional ports were required. RPRDG showed longer operation time than CLDG (188.4 min vs. 166.2 min, p < 0.001) and similar operation time with CRDG (183.1 min, p = 0.315). The blood loss was 35.4 ml for RPRDG, 85.2 ml for CLDG (p < 0.001), and 41.2 ml for CRDG (p = 0.33). The numbers of retrieved lymph nodes were 50.5 for RPRDG, 43.9 for CLDG (p = 0.003), and 55.0 for CRDG (p = 0.055). Postoperative maximum C-reactive protein levels were 96.8 mg/L for RPRDG, 87.8 mg/L for CLDG (p = 0.454), and 81.9 mg/L for CRDG (p = 0.027). Learning curve analysis indicated that the overall operation time of RPRDG stabilized at 180 min after 21 cases. The incidence of major postoperative complications did not differ among groups. RPRDG for gastric cancer is a feasible and safe alternative to conventional minimally invasive surgery. Notwithstanding, this procedure failed to reduce postoperative inflammatory responses.

## Introduction

With the accumulation of personal and institutional experiences and with the development of laparoscopic instruments since the first reported laparoscopic gastrectomy with lymph node dissection^1^, experienced surgeons have successfully shifted to more advanced procedures, such as reduced-port or single-incision laparoscopic gastrectomy, for gastric cancer^2–6^. Reduced-port laparoscopic gastrectomy was developed in expectation of better short-term surgical outcomes by reducing surgical insults to the patients. However, other than better cosmetic results, its outcomes appear to be similar to those of conventional laparoscopic surgery^3,5^, and surgeons have raised concerns for its technical and oncology safety due to a limited number of access points that leads to intra- and extracorporeal collisions^7,8^.

As an alternative to reduced-port laparoscopic gastrectomy, we previously introduced reduced-port totally robotic gastrectomy^9,10^. In our initial experience, we found that this procedure alleviated many of the ergonomic and technical difficulties encountered when performing reduced-port laparoscopic gastrectomy. Moreover, applying the reduced-port totally robotic procedure, we were able to successfully conduct D2 lymph node dissection and perform technically challenging anastomosis, such as intracorporal delta-shape gastroduodenostomy, without compromising short-term surgical outcomes. However, there were limitations to our reports on this initial experience because they involved non-comparative analysis and small sample sizes.

Thus, in this study, we sought to investigate the short-term outcomes of our initial 100 consecutive cases of reduced-port totally robotic distal subtotal gastrectomy, comparing them with the short-term outcomes of conventional laparoscopic and robotic distal subtotal gastrectomy.

## Results

### Patient characteristics

Patient characteristics are outlined in Table 1. Mean age was 54.5 years in the RPRDG group, 62.0 years in the CLDG group (p < 0.001) and 57.2 years in the CRDG group (p = 0.059). Mean BMI values were 23.8, 23.5, and 23.6, respectively, and differences therein showed no statistical significance in each comparison (RPRDG vs. CLDG; p = 0.544, RPRDG vs. CRDG; p = 0.689). While the CLDG group showed a higher proportion of patients with worse ASA score (p = 0.001) than the RPRDG group, ASA scores for CRDG and RPRDG were similar (p = 0.383). Clinical T, N classification and tubular, circular tumor location were similar between the RPRDG and CLDG groups (p = 0.216, 0.422, 0.378, and 0.919, respectively) and between the RPRDG and CRDG groups (p = 0.852, 1, 0.18, and 0.794, respectively).

**Table 1:** Patient characteristics.

Variables	Reduced robotic (*n* = 100)	Conventional laparoscopic (*n* = 261)	*p* value	Conventional robotic (*n* = 241)	*p* value
Age (years)	54.5 ± 11.4	62.0 ± 11.5	< 0.001	57.2 ± 12.1	0.059
**Sex**			0.733		0.505
Male	59 (59.0)	147 (56.3)		131 (54.4)	
Female	41 (41.0)	114 (43.7)		100 (45.6)	
**ASA score**			0.001		0.383
1	24 (24.0)	39 (14.9)		63 (26.1)	
2	62 (62.0)	129 (49.4)		129 (53.5)	
3	14 (14.0)	86 (33.0)		47 (19.5)	
4	0 (0)	7 (2.7)		2 (0.8)	
BMI, kg/m^2^	23.8 (2.7)	23.5 (3.1)	0.544	23.6 (2.9)	0.689
**Clinical T classification**^**a**^			0.216		0.852
cT1	74 (74.0)	212 (81.2)		185 (76.8)	
cT2	21 (21.0)	41 (15.7)		48 (19.9)	
cT3	4 (4.0)	8 (3.1)		7 (2.9)	
cT4	1 (1.0)	0 (0.0)		1 (0.4)	
**Clinical N classification**^**a**^			0.422		> 0.99
N0	89 (89.0)	241 (92.3)		214 (88.8)	
N1	11 (11.0)	20 (7.7)		27 (11.2)	
**Tubular tumor location**			0.378		0.18
Middle third	67 (67.0)	160 (61.3)		141 (58.5)	
Lower third	33 (33.0)	101 (38.7)		100 (41.5)	
**Circular tumor location**			0.919		0.794
Lesser curvature	33 (33.0)	94 (36.0)		88 (36.5)	
Greater curvature	19 (19.0)	56 (21.5)		49 (20.3)	
Anterior wall	21 (21.0)	47 (18.0)		37 (15.4)	
Posterior wall	26 (26.0)	62 (23.8)		65 (27.0)	
Circular	1 (1.0)	2 (0.8)		2 (0.8)	

Values are shown mean ± standard deviation or n(%).

^a^Clinical stages are according to the AJCC 8th staging system.

BMI, body mass index; ASA score, America Society of Anesthesiologist score.

### Operative and pathologic outcomes (Table 2)

**Table 2:** Operative and pathologic outcomes.

Variables	Reduced robotic (*n* = 100)	Conventional laparoscopic (*n* = 261)	*p* value	Conventional robotic (*n* = 241)	*p value*
Open or laparoscopic conversion	0 (0)	0 (0)	–	0 (0)	–
Lymph node dissection			0.066		0.804
D1	0 (0.0)	0 (0.0)		1 (0.4)	
D1 +	73 (73.0)	215 (82.4)		177 (73.4)	
D2	27 (27.0)	46 (17.6)		63 (26.1)	
Reconstruction			0.005		0.003
BI	91 (91.0)	198 (75.9)		181 (75.1)	
BII	8 (8.0)	52 (19.9)		45 (18.7)	
Roux-en-Y	1 (1.0)	11 (4.2)		15 (6.2)	
Omentectomy			0.908		0.679
Partial	96 (96.0)	248 (95.0)		227 (94.2)	
Total	4 (4.0)	13 (5.0)		14 (5.8)	
Combined resection			0.47		0.145
No	94 (94.0)	230 (88.1)		236 (97.9)	
Gallbladder	4 (4.0)	21 (8.0)		2 (0.8)	
Colon	0 (0.0)	2 (0.8)		1 (0.4)	
Ovary	0 (0.0)	2 (0.8)		0 (0.0)	
Others	2 (2.0)	6 (2.3)		2 (2.0)	
Operation time (min)	188.4 ± 40.3	166.2 ± 58.4	< 0.001	183.1 ± 50.7	0.315
Blood loss (ml)	35.4 ± 40.8	85.2 ± 95.2	< 0.001	41.2 ± 66.4	0.33
No. retrieved lymph node	50.5 ± 20.2	43.9 ± 18.1	0.003	55.0 ± 19.6	0.055
Median (range) No. of retrieved lymph node	44.5 (17–119)	42.0 (8–156)	0.011	53.0 (16–130)	0.018
No. metastatic lymph node	0.7 ± 2.4	0.6 ± 1.9	0.601	1.3 ± 6.2	0.187
Proximal margin (mm)	40.3 ± 22.7	46.4 ± 28.1	0.034	41.1 ± 26.5	0.782
Proximal margin involvement	1 (1.0)	0 (0.0)	0.618	0 (0.0)	0.649
Distal margin (mm)	54.3 ± 36.4	58.3 ± 36.0	0.349	63.0 ± 36.8	0.047
Distal margin involvement	0 (0.0)	0 (0.0)	–	0 (0.0)	–
Histologic type			0.007		0.079
Well differentiated	14 (14.0)	24 (9.2)		22 (9.1)	
Moderately differentiated	17 (17.0)	98 (37.5)		73 (30.3)	
Poorly differentiated	43 (43.0)	77 (29.5)		78 (32.4)	
Mucinous	0 (0.0)	1 (0.4)		1 (0.4)	
Signet ring cell	23 (23.0)	55 (21.1)		63 (26.1)	
Others	3 (3%)	6 (2.3)		4 (1.7)	
pTNM stage^a^			0.887		0.816
IA	74 (74.0)	203 (77.8)		179 (74.3)	
IB	10 (10.0)	24 (9.2)		20 (8.3)	
IIA	5 (5.0)	14 (5.4)		12 (5.0)	
IIB	3 (3.0)	8 (3.1)		11 (4.6)	
IIIA	6 (6.0)	9 (3.4)		9 (3.7)	
IIIB	2 (2.0)	3 (1.1)		7 (2.9)	
IIIC	0 (0.0)	0 (0.0)		3 (1.2)	

Values are shown mean ± standard deviation or n(%).

^a^Stages are according to the 8th AJCC TNM staging system.

There were no open or laparoscopic conversions or additional port insertions during any procedure. D2 lymph node dissection rate was higher in the RPRDG group (27%) than in the CLDG group (17.6%), although the difference was not statistically significant (p = 0.066). Between the RPRDG and CRDG groups, D2 lymph node dissection rates were similar (26.1% in CRDG, p = 0.804). Reconstruction was primarily achieved by BI anastomosis in all groups, although the rate was significantly higher in the RPRDG group (91.0%) than in the CLDG (75.9%, p = 0.005) and CRDG groups (75.1%, p = 0.003). Operation time was significantly longer for RPRDG than for CLDG (188.4 min vs. 166.2 min, p < 0.001) and was similar between RPRDG and CRDG (183.1 min, p = 0.315). Estimated blood loss in the RPRDG group was significantly less than that in the CLDG group (35.4 ml vs. 85.2 ml, p < 0.001), but similar to that in the CRDG group (41.2 ml, p = 0.33). The mean number of retrieved lymph nodes in the RPRDG group was significantly larger than that in the CLDG group (50.5 vs. 43.9, p = 0.003), but similar to that in the CRDG group (55.0, p = 0.055). The median number of retrieved lymph nodes in the RPRDG group was still larger than that in the CLDG group (44.5 vs. 42.0, p = 0.011), however significantly smaller to that in the CRDG group (53.0, p = 0.018). One patient in the RPRDG group underwent R1 resection due to a positive proximal margin, although frozen section biopsy showed the proximal margin involvement to be false negative.

### Postoperative outcomes (Table 3)

**Table 3:** Postoperative outcomes.

Variables	Reduced robotic (*n* = 100)	Conventional laparoscopic (*n* = 261)	*p* value	Conventional robotic (*n* = 241)	*p* value
Bowel function recovery (days)	3.3 ± 0.8	3.3 ± 1.1	0.744	3.2 ± 0.7	0.27
Soft diet (days)	4.9 ± 3.5	4.7 ± 2.1	0.587	4.0 ± 0.4	0.02
Hospital stay (days)	6.6 ± 4.5	6.6 ± 3.5	0.947	5.4 ± 1.1	0.008
Maximum WBC (10^3/µL)	13.7 ± 3.3	13.1 ± 3.3	0.053	13.0 ± 3.5	0.106
Minimum Hemoglobin (g/dL)	11.3 ± 1.4	11.0 ± 1.4	0.213	11.4 ± 1.4	0.742
Minimum Albumin (g/dL)	3.4 ± 0.3	3.3 ± 0.3	0.164	3.4 ± 0.3	0.367
Maximum C-reactive protein (mg/L)	96.8 ± 57.4	87.8 ± 54.2	0.454	81.9 ± 56.1	0.027
Major complication^a^			1		1
No	99 (99.0)	259 (99.2)		238 (98.8)	
Yes	1 (1.0)	2 (0.8)		3 (1.2)	
Readmission due to major complication^b^			–		0.629
No	100 (100.0)	251 (99.6)		238 (98.8)	
Yes	0 (0.0)	1 (0.4)		3 (1.2)	
Mortality^b^	0 (0.0)	0 (0.0)		2 (0.8)	

Values are shown mean ± standard deviation or n(%).

^a^Major complication was defined as Clavien–Dindo complication grade III or higher.

^b^Within 90 days after the operation.

**Figure 1:**
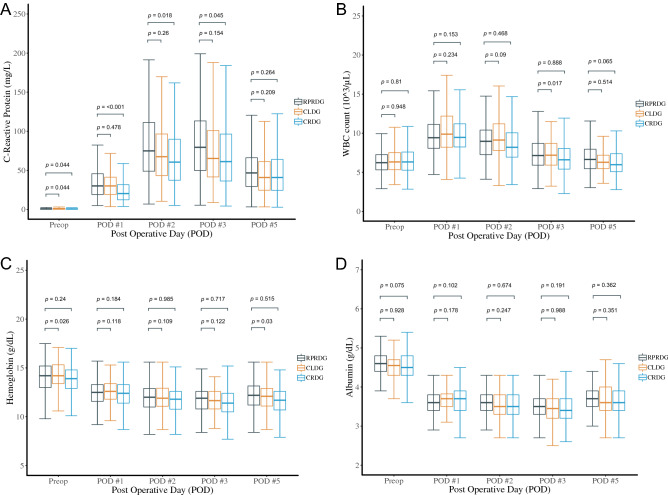
Changes in postoperative laboratory results until postoperative day 5 for reduced-port robotic, conventional laparoscopic, and conventional robotic distal subtotal gastrectomy. (**A**) C-reactive protein; (**B**) white blood cell counts; (**C**) hemoglobin; (**D**) albumin.

Resumption of a soft diet and the length of hospital stay in the RPRDG group did not differ from those in the CLDG group (4.9 days vs. 4.7 days, p = 0.587 and 6.6 days vs. 6.6 days, p = 0.947, respectively), but were longer than those in the CRDG group (4.0 days, p = 0.02 and 5.4 days, p = 0.008, respectively). Postoperative laboratory test results were similar, except for maximum C-reactive protein (CRP), which was greater in the RPRDG group than in the CRDG group (96.8 vs. 81.9, p = 0.027). Postoperative CRP, WBC, hemoglobin, and albumin levels among the study groups at POD 1, 2, 3, and 5 are depicted in Fig. 1. Although the RPRDG group showed the highest levels of CRP among the three groups, they became similar to those of the other procedures at POD 5. Also, we found no significant difference between RPRDG and other groups in postoperative WBC, hemoglobin, or albumin levels. Rates of in-hospital major complications and readmission associated with major surgical complication were similar among the groups. In the RPRDG group, one grade IIIb, major in-hospital complication occurred due to intestinal obstruction that required re-operation; there were no readmissions due to major complications. In the CLDG group, two major in-hospital complications of postoperative pneumonia and intraabdominal fluid collection were recorded. One patient in the CLDG group was readmitted on POD 19 due to anastomosis leakage that was treated with percutaneous drainage. In the CRDG group, there were three major in-hospital complications of postoperative cardiac arrhythmia, wound complication, and sudden cardiac arrest. Also, three patients treated with CRDG were readmitted due to major complications of duodenal stump leakage, gastric ulcer bleeding, and intraabdominal bleeding. There were two mortality cases, which occurred only in the CRDG group: one case involved sudden cardiac arrest of suspected cardiac origin on POD 7, and the other involved hypovolemic shock due to intraabdominal bleeding at POD 26 after readmission.

### Learning curve analysis for RPRDG

**Figure 2:**
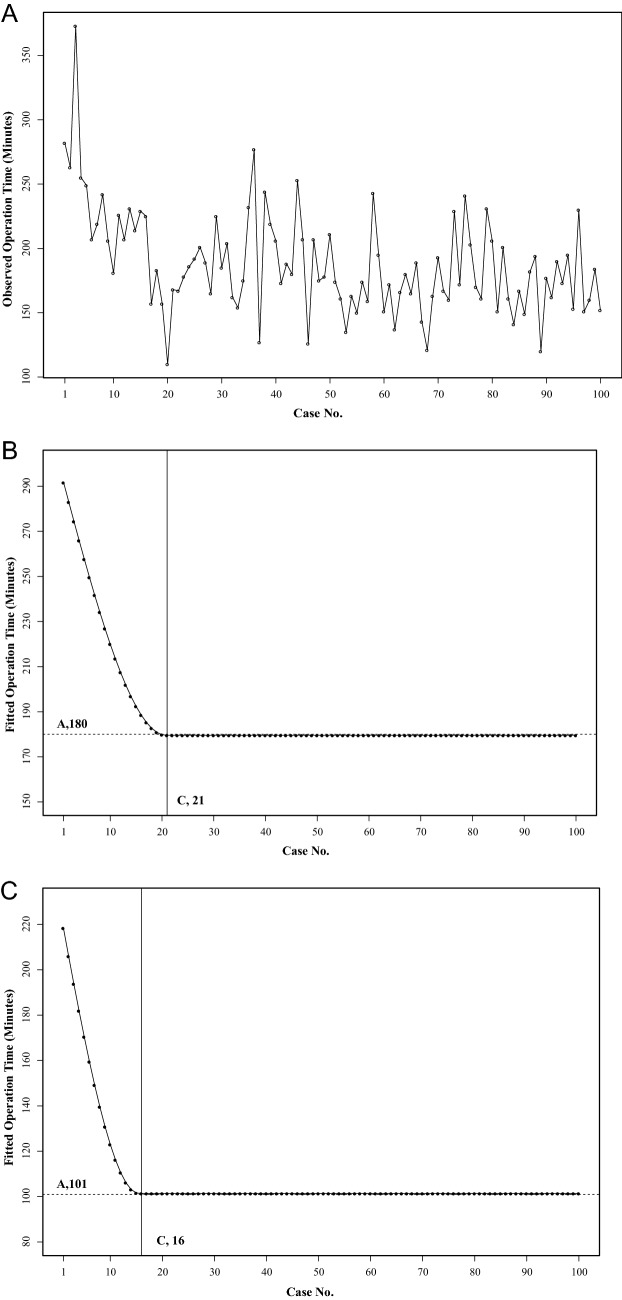
Learning curve analysis of reduced-port totally robotic gastrectomy. (**A**) Actual operation times for reduced-port totally robotic distal subtotal gastrectomy. (**B**) Fitted operation times by a nonlinear regression model for reduced-port totally robotic distal subtotal gastrectomy (A, stable operation time; C, converged case number). (**C**) Fitted operation time after adjusting for confounding variables of reduced-port totally robotic distal subtotal gastrectomy (A, stable operation time; C, converged case number).

The actual operation times for RPRDG are drawn in Fig. 2A. Initial operation times ranged from 200 to 300 min, which decreased to less than 200 min at around 20 cases. The results of fitted operation time analysis with the nonlinear regression model in Eq. (1). are plotted in Fig. 2B. The overall operation time stabilized at 180 min after 21 cases, a reduction of 120 min from the first case of RPRDG. We also used a nonlinear regression model with linear combinations (Eq. 2) to adjust for confounding factors affecting the operation time of RPRDG. According to this analysis, the fitted operation time stabilized at 101 min after 16 cases, for a reduction of 130 min (Fig. 2C). Among the confounding factors accounted for in the fitted analysis, age (0.48 min), D2 lymph node dissection (42.08 min, reference to D1 +), and BII or RY reconstruction (9.58 min, reference to BI) were statistically significant (eTable 1).

## Discussion

In this study, we demonstrated the technical feasibility and safety of RPRDG for gastric cancer. Over our initial 100 consecutive procedures, we have not experienced any open or laparoscopic conversion or additional port insertion due to technical difficulties. Also, learning curve analysis of our initial 100 RPRDG procedures showed an acceptable stabilized operation time of 180 min within 21 cases, which was faster than what we encountered for laparoscopic reduced-port procedures^6,11–13^. Other short-term surgical outcomes of RPRDG were comparable to those for CLDG performed by the same surgeon and for CRDG performed by another surgeon with considerable experience in this field. Notwithstanding, RPRDG failed to facilitate improvements in postoperative inflammatory markers, compared with conventional minimally invasive surgery groups.

Unlike conventional laparoscopic gastrectomy, which has a reported learning curve of more than 50 cases^14–16^, robotic gastrectomy has a learning curve of 10–20 cases^12,17^. Recent reports have shown that reduced-port or single-incision laparoscopic gastrectomy conducted by experienced laparoscopic surgeons reaches stabilized operation times after 30–50^6,11–13^. In our learning curve analysis, RPRDG showed results similar to those for conventional robotic gastrectomy^12^. We presumed that this was because RPRDG is able to reduce the technical difficulties with reduced-port laparoscopic gastrectomy, especially in intra- and extra-corporeal collisions, which are the main hindrances in this surgery. However, when we analyzed factors influencing the operation time of RPRDG, older age, lymph node dissection greater than D1 + , and anastomosis other than BI were related to longer operation times during the RPRDG procedure. These results were different from a previous report^12^, and imply that limited access and a narrow range of motion for robotic instruments in reduced-port gastrectomy still exist, even with the current robotic surgical system.

In this study, objective postoperative inflammatory markers, such as CRP and WBC count^18^, did not differ among the surgery groups. We hypothesized that if RPRDG could decrease injury to the abdominal wall by reducing the number of incisions, then inflammatory markers during the postoperative period might be lowered, compared with conventional laparoscopic or robotic surgery. According to our analysis, however, maximum CRP and WBC counts for RPRDG were no better than those for other conventional procedures. Moreover, maximum CRP was higher in the RPRDG group than in the other surgery groups in the earlier postoperative period, although they were similar at the time of discharge. Considering other surgical variables that could affect the inflammatory process, for instance, D2 lymph node dissection, the rates of which were similar among the groups, we suspect that a longer operation time and the 2.5-cm incision that was made at the beginning of the procedure were the primary causes of these results. Also, similar results were described in a prior study of reduced-port laparoscopic gastrectomy^3^. Nevertheless, postoperative morbidity and mortality may not be affected by a slight increase in inflammatory markers in the early post-operative period, and we have not experienced any incisional hernia in the infraumbilical or port incisions during follow up after RPRDG.

RPRDG was designed to emulate CRDG. In terms of surgical outcomes, operation time, blood loss, and the number of retrieved lymph nodes were similar between the two procedures. Meanwhile, a longer operation time for robotic gastrectomy, compared with laparoscopic surgery, which has been reported in previous studies^19,20^, was noted in the current study. However, compared with CLDG, RPRDG was able to decrease estimated blood loss by about 50 ml and to retrieve 10 more lymph nodes. In terms of oncologic safety, we consider that spending 20 more minutes to lose less blood and to retrieve more lymph nodes may be acceptable. Accordingly, we deemed RPRDG to be comparable to other conventional procedures.

This is the first comparative study of 100 consecutive cases of reduced-port robotic distal subtotal gastrectomy for gastric cancer. Herein, we were able to demonstrate the feasibility and safety of our reduced-port robotic procedure, which showed a shorter learning curve and comparable short-term outcomes with conventional minimally invasive surgeries. This study did, however, have a couple of limitations. First, it was a study with a retrospective design. Second, RPRDG was only compared with conventional robotic surgery performed by another surgeon, since the operator of RPRDG has continued to perform all robotic procedures in a reduced-port fashion since its implementation. Third, we could not compare RPRDG with another reduced-port procedure because reduced-port laparoscopic gastrectomy is not performed in our institution. Finally, although we analyzed postoperative inflammatory markers of the procedures, we did not assess postoperative quality of life and cosmetic satisfaction with the procedures as evaluated by the patients. Due to the limitations listed above, there may be issues in generalizing the results of the current study. In particular, the results of comparing reduced-port robotic gastrectomy with conventional surgery by the experts in the field of robotic gastrectomy may be difficult to apply in countries other than Korea.

In conclusion, while this study only describes our initial experience, RPRDG showed short-term outcomes comparable to conventional laparoscopic and robotic gastrectomy. Although operation times were a bit longer for RPRDG, they were acceptable, and the number of cases to overcome the learning curve was only around 20 cases, after which operation times became shorter. However, we did not observe decreased surgery-related stress for RPRDG, compared to conventional procedures, and further studies are required to justify the real benefits of RPRDG procedure over laparoscopic reduced-port surgery. Prospective trials are needed to provide more confirmative results on the short-term and long-term outcomes of various types of reduced-port robotic surgeries in comparison with conventional minimally invasive surgeries.

## Patients and method

### Patients

We retrospectively reviewed a prospective collected database of gastric cancer patients treated at Severance Hospital, Yonsei University College of Medicine. Between February 2016 and September 2018, 362 patients underwent distal subtotal gastrectomy for gastric cancer with a minimally invasive approach by a single surgeon. There were 100 consecutive reduced-port totally robotic surgery cases and 261 conventional laparoscopic surgery cases. One patient during the study period underwent conventional five-port robotic distal subtotal gastrectomy combined with radical prostatectomy for prostate cancer and was excluded from analysis. During the same period, another surgeon (WJH) performed 241 conventional robotic distal subtotal gastrectomies.

The indication for minimally invasive surgery was early gastric cancer beyond the indication of endoscopic submucosal dissection or serosa negative advanced gastric cancer. Preoperative staging was assessed by endoscopy with or without endoscopic ultrasound and by abdomino-pelvic computed tomography. All patients provided informed consent for surgery, including available minimally invasive surgery procedures. In cases in which serosal involvement was suspected during preoperative evaluation, minimally invasive surgery was carried out at the patient’s request. This retrospective study was approved by the Institutional Review Board of Severance Hospital, Yonsei University College of Medicine (4-2019-0528). All methods were carried out in accordance with relevant guidelines and regulations.

### Surgical procedure

Details on the surgical procedure of reduced-port totally robotic distal subtotal gastrectomy (RPRDG) have been described previously^9,10^. In RPRDG, an overturned SINGLE-SITE port (Intuitive Surgical, Sunnyvale, CA, USA) was placed via a 25-mm midline incision below the umbilicus. A camera, a semirigid Cadiere forceps, and an assistant port were equipped via the openings in the SINGLE-SITE port. An additional two ports were placed on both sides of the abdomen for rigid instruments. An 8-mm port was placed in the right abdomen for equipping ultrasonic shears, and a 12-mm port was placed in the left abdomen for introducing Maryland bipolar forceps and staplers during anastomosis. During BI anastomosis, endolinear staplers or robotic ENDOWRIST staplers (Intuitive Surgical, Sunnyvale, CA, USA) were inserted through the left port, and for BII or RY anastomosis, we converted the 8-mm port in the right abdomen to a 12-mm port for inserting and applying the staplers.

The standard procedure for conventional robotic distal subtotal gastrectomy (CRDG), which utilizes a total of five ports and four robotic arms, has been published elsewhere^21^. A camera was inserted through a port positioned below the umbilicus. Cadiere forceps were inserted through an 8-mm port on the right lateral side of the abdomen, and ultrasonic shears were inserted through another 8-mm port on the middle point between the right lateral port and umbilicus. Another 8-mm port on the left lateral side of the abdomen was placed for Maryland bipolar forceps, and a 12-mm port was placed on the middle point between the left lateral port and umbilicus for an assistant. Staplers for anastomosis were inserted from the 12-mm port by the assistant for BI anastomosis or from the right middle side after extension of the 8-mm port to a 12-mm port for BII or RY anastomosis.

Conventional laparoscopic distal subtotal gastrectomy (CLDG) was performed with two 5-mm ports on both sides of the upper abdomen, two 12-mm ports on both sides of the abdomen, and one 12-mm camera port just below the umbilicus. During the laparoscopic surgery, the operator could freely choose among the ports for employing various instruments, although ultrasonic shears were primarily inserted via the right middle port. Also, staplers were inserted in the 12-mm ports in the right or left side as appropriate for BI, BII, or RY reconstruction. Laparoscopic reduced-port surgery, including single-incision gastrectomy, for gastric cancer was not performed in our institution.

### Postoperative management and data collection

Postoperative management plans for stomach cancer patients were similar among operative procedures and surgeons. On postoperative day (POD) 2, patients without serious complications were allowed sips of water. A liquid diet and soft diet specially designed for patients undergoing gastrectomy was supplemented in POD 3 and POD 4, respectively. Routine laboratory tests for hematologic, routine chemistry, and inflammatory markers were checked on POD 1, 2, 3, and 5. On POD 5, we recommended discharge for patients with normal laboratory test results who well adapted to the soft diet. Postoperative complications were documented in our database weekly after a meeting with surgical faculty and residents who reviewed and classified the complications according to Clavien-Dindo classification. Readmission within 90 days of operation that was found to be related to surgical complications was also recorded in the database.

### Statistical analysis

Patient characteristics and operative related variables were tested to check for differences among the study groups: we compared the RPRDG group versus the CRDG group and the RPRDG group versus the CLDG group. Differences between groups were analyzed using Student’s t-test, and descriptive statistics are represented as means ± SDs. Categorical variables with counts and percentages were analyzed by the chi-square test. For categorical variables in which more than 20 percent of all cells had expected counts fewer than 5, Fisher’s exact test was performed.

For learning curve analysis, as in a previous study^12^, we fitted the nonlinear regression model below to investigate the relationship between operation time ($\mathrm{t}$, the number of operations) and surgery experience (${y}_{t}$, operation time at t-th operation):

1 $${y}_{t}=a+ {\varepsilon }_{t}+ \left\{\begin{array}{c}{c}_{1}\times \left(1-\frac{3t}{2{c}_{2}}+\frac{1}{2}{\left(\frac{t}{{c}_{2}}\right)}^{3}\right), t<{c}_{2},\\ 0, t\ge {c}_{2}.\end{array}\right.$$

The model states that, as operations accumulate, starting from an initial time ($a+{c}_{1}$), the mean operation time decreases nonlinearly until it reaches a stable time ($a$) after a number (${c}_{2}$) of operations. This learning feature is well-represented by the learning curve model in (Eq. 1). Also, we fitted the model by adding covariates of interest, such as sex, age, BMI, extent of lymph node dissection, and reconstruction type, that could affect operation time. These covariates were described in a previous study^22^ as an extended model:

2 $${y}_{t}=a+ {\beta }_{1}{x}_{1t}+\cdots +{\beta }_{p}{x}_{pt}+{\varepsilon }_{t}+ \left\{\begin{array}{c}{c}_{1}\times \left(1-\frac{3t}{2{c}_{2}}+\frac{1}{2}{\left(\frac{t}{{c}_{2}}\right)}^{3}\right), t<{c}_{2},\\ 0, t\ge {c}_{2}.\end{array}\right.$$

Statistical significance was set at a *P* value < 0.05 for all analyses, and all analyses were conducted using R version 3.5.1 (The R Foundation for Statistical Computing, Vienna, Austria).

## Supplementary information

Supplementary information.

## Data Availability

The datasets generated and analyzed during the current study are available from the corresponding author on reasonable request.
