# Supplementary material for: Reduced-port totally robotic distal subtotal gastrectomy for gastric cancer: 100 consecutive cases in comparison with conventional robotic and laparoscopic distal subtotal gastrectomy
Source: Sci Rep. 2020 Sep 29;10:16015. doi: 10.1038/s41598-020-73118-9 (PMC7524794; doi:10.1038/s41598-020-73118-9)
Supplement: Supplementary file 1 — Supplementary information. [file 41598_2020_73118_MOESM1_ESM.docx]

**Reduced-port totally robotic distal subtotal gastrectomy for gastric cancer: 100 consecutive cases in comparison with conventional robotic and laparoscopic distal subtotal gastrectomy**

Won Jun Seo^1^, Taeil Son^2,3,4*^, Hyejung Shin^5^, Seohee Choi^2^, Chul Kyu Roh^6^, Minah Cho^2,3^, Hyoung-Il Kim^2,3,4^, Woo Jin Hyung^2,3^,

^1^Department of Surgery, Korea University College of Medicine, Seoul, Korea

^2^Department of Surgery, Yonsei University College of Medicine, Seoul, Korea

^3^Gastric Cancer Center, Yonsei Cancer Center, Yonsei University Health System, Seoul, Korea

^4^Robot and MIS Center, Severance Hospital, Yonsei University Health System, Seoul, Korea

^5^Biostatistics Collaboration Unit, Department of Biomedical Systems Informatics, Yonsei University College of Medicine, Seoul, Korea

^6^Department of Surgery, Ajou University School of Medicine, Suwon, Korea

**Supplementary information**

**eTABLE 1.** Estimates of learning model parameters and effects of confounding factors affecting operation time for reduced-port totally robotic distal subtotal gastrectomy

| Variables | Estimate (SE) | *p* value |
| --- | --- | --- |
| Stable operation time (a) | 101.39 ± 25.39 | 0.001 |
| Reduced operation time (c1) | 129.52 ± 18.41 | <0.001 |
| Case no. converged (c2) | 15.51 ± 2.48 | <0.001 |
| Gender (reference = “female”) | 3.53 ± 5.40 | 0.52 |
| Age | 0.48 ± 0.23 | 0.042 |
| BMI | 1.63 ± 1.01 | 0.108 |
| D2 Lymph node dissection (reference = “D1+”) | 42.08 ± 6.04 | <0.001 |
| BII or RY reconstruction (reference = “BI”) | 9.58 ± 2.28 | 0.025 |

Abbreviation: BI, Billroth I (gastroduodenustomy); BII, Billroth II (gastrojejunostomy); RY (Roux-en-Y gastrojejunostomy)
